# Tracking the origin of simultaneous endometrial and ovarian cancer by next-generation sequencing – a case report

**DOI:** 10.1186/s12885-017-3054-6

**Published:** 2017-01-19

**Authors:** Nadejda Valtcheva, Franziska M. Lang, Aurelia Noske, Eleftherios P. Samartzis, Anna-Maria Schmidt, Elisa Bellini, Daniel Fink, Holger Moch, Markus Rechsteiner, Konstantin J. Dedes, Peter J. Wild

**Affiliations:** 1 Institute of Pathology, University Hospital Zurich, Zurich, Switzerland; 2 Department of Gynaecology, University Hospital Zurich, Zurich, Switzerland

**Keywords:** Endometrial cancer, Ovarian cancer, Next-generation sequencing, Metastasis, Case report

## Abstract

**Background:**

Endometrioid adenocarcinoma of the uterus and ovarian endometrioid carcinoma share many morphological and molecular features. Differentiation between simultaneous primary carcinomas and ovarian metastases of an endometrial cancer may be very challenging but is essential for prognostic and therapeutic considerations.

**Case Presentation:**

In the present case study of a 33 year-old patient we used targeted amplicon next-generation re-sequencing for clarifying the origin of synchronous endometrioid cancer of the corpus uteri and the left ovary. The patient developed a metachronous lung metastasis of an endometrioid adenocarcinoma four years after hyster- and adnexectomy, vaginal brachytherapy and treatment with the synthetic steroid tibolone. Removal of the metastasis and megestrol treatment for seven years led to a complete remission.

A total of 409 genes from the Ampliseq Comprehensive Cancer Panel (Ion Torrent, Thermo Fisher) were analysed by next generation sequencing and mutations in 10 genes, including *ARID1A*, *CTNNB1*, *PIK3CA* and *PTEN* were identified and confirmed by Sanger sequencing. Primary endometrial as well as ovarian cancer showed an identical mutational profile, suggesting the presence of an ovarian metastasis of the endometrial cancer, rather than a simultaneous endometrial and ovarian cancer. The metachronous lung metastasis showed a different mutational profile compared to the primary cancer. Immunohistochemical staining of the corresponding proteins suggested that the tumour development was driven by alterations in the protein function rather than by changes of the protein abundance in the cell.

**Conclusions:**

Our results have demonstrated next generation sequencing as a valuable tool in the differentiation of synchronous primary tumours and metastases, which has an important impact on the clinical decision making process. Similar to breast cancer, targeted therapies based on mutational tumour profiling will become increasingly important in endometrial and ovarian cancer. In summary, our results support the usage of next generation sequencing as a supplementary diagnostic tool, assisting in personalized precision medicine.

**Electronic supplementary material:**

The online version of this article (doi:10.1186/s12885-017-3054-6) contains supplementary material, which is available to authorized users.

## Background

Endometrioid adenocarcinoma of the uterus and ovarian endometrioid carcinoma share many morphological and molecular features [1, 2]. Coincidence of both primary tumours is a relatively common event which occurs in about 10% of patients with ovarian carcinoma and in about 5% with endometrial carcinoma [3]. When simultaneous uterine and ovarian carcinomas show the same histology and differentiation, determination of the primary origin may be difficult. In case of independent primary tumours, this might be due to the several common risk factors shared by the two malignancies or due to germline alterations that predispose to the development of simultaneous cancers. In most cases, however, one of the endometrioid type carcinomas is the primary tumour that gives rise to a metastatic lesion. It is also not excluded that both carcinomas might originate from a common endometrial epithelial precursor cell of origin, a theory that can explain the histological and genetic similarities of the lesions [4]. Distinguishing between two independent lesions versus one primary and one metastatic lesion is extremely difficult, nevertheless important for the prognosis and treatment of the patient [5].

A novel powerful tool to identify common genetic alterations specific for a cancer entity and to provide information on the tumour clonal evolution is next-generation sequencing (NGS) [6]. To date, the knowledge of the mutational landscape of many different tumours including endometrial and ovarian cancer has increased throughout the last 10 years. TCGA has published data from whole exome sequencing of a large number of both gynaecological malignancies [1, 2]. Furthermore, studies have been reported on endometrial and ovarian carcinoma using two different cohorts – one including patients with endometrial endometrioid carcinomas and one consisting of ovarian endometrioid carcinomas [4]. The authors suggest that although the same genes are affected in both cancers the mutation patterns differ possibly due to the different microenvironment in the ovary and the uterus. This offers a possibility to determine the origin of simultaneously identified endometrial and ovarian endometrioid lesions. While these reports contribute to the big picture of what are the most common aberrations in the corresponding tumour type and beyond doubt profit from the large number of cases investigated, single patients with synchronously diagnosed endometrial and ovarian cancer can shed light on the direct tumour evolution. Although NGS is broadly used in preclinical research, thus providing the possibility to unravel genetic alterations and potential therapeutic targets, in the clinical setting current diagnostics and treatment of metastatic endometrial and ovarian cancer is still mainly based on radiological and conventional histological results as well as chemotherapy combination regimens.

In the present case report we investigated the mutation pattern in a 33-year-old female patient diagnosed with synchronous endometrioid adenocarcinoma of the corpus uteri and the left ovary. The patient developed a lung metastasis of an endometrioid type adenocarcinoma four years after laparoscopic hysterectomy, vaginal brachytherapy and tibolon therapy. Based on histological findings, it is difficult to decide whether the gynaecological carcinomas are two independent primary carcinomas occurring in parallel. Furthermore, it is almost impossible to recognize the origin of the lung mass. Thus, we employed targeted NGS of 409 tumour-related genes (Ion Ampliseq Comprehensive Cancer Panel, Thermo Fisher) in order to identify the relation between the two carcinomas and the source of the distant metastasis. Moreover, we were interested in the genetic fingerprint of the metastasis to clarify the recurrence-free survival of the patient after its removal and megestrol treatment for seven years. Finally, we looked for possible germline mutations from blood samples of the patient that could explain the early onset of the disease.

## Methods

### Clinical history and pathological findings

A 33-year-old woman, who has undergone a diagnostic curettage due to hypermenorrhoea and menorrhagia, was diagnosed with endometrial endometrioid adenocarcinoma of moderate differentiation and with squamous components. Subsequently, a laparoscopic hysterectomy with pelvic washing, bilateral adnexectomy and pelvic lymphadenectomy was performed. In the hysterectomy, well-differentiated residual cancer was found with infiltration less than one half of the myometrium, consistent with stage pT1a.

The tumour tissue showed high expression of oestrogen and progesterone receptors (both 100%, Fig. 3). Unexpectedly, a synchronous well differentiated endometrioid adenocarcinoma with squamous components of the left ovary was detected. The tumour mass was confined to the ovary according to stage pT1a. The contralateral ovary showed no pathological alterations. All pelvic lymph nodes (*n* = 20) were free of tumour. In summary, the former pathological TNM classification for the corpus uteri was pT1a pN0 (0/20) G2 (FIGO stage IA) and for the ovary pT1a pN0 G1 (FIGO stage IA). Vaginal brachytherapy of 4x5 Gy was administered because of a G2 tumour and the quite young age of the patient. After treatment with tibolon (2.5 mg/day) for 4 years, the patient presented with chest pain in the right upper thorax. Through CT-scan and the following thoracoscopic resection of the right upper lobe of the lung, a metastatic endometrioid adenocarcinoma was removed. Treatment with megestrol (160 mg/day) was started. In addition to the clinical check-up, a CT-scan was performed each year. The latest PET–CT scan after 7 years being treated with megestrol acetate showed no evidence of tumour recurrence. Informed consent was obtained and the study has been approved by the local ethics committee (KEK-ZH-No. 2010–0358).

### Immunohistochemical analysis

Tumour tissue sections were immunohistochemically investigated with the following antibodies: oestrogen receptor (SP1 Ventana-Roche, prediluted), progesterone receptor (1E2, Ventana-Roche, prediluted), mismatch repair proteins as MSH2 (25D12, Novocastra Lab Ltd., 1:100), MSH6 (BD Biosciences 1:500), MLH1 (G168-15, PharMingen Becton Dickinson 1:100), PMS2 PharMingen BD 1:300), PTEN (6H2.1 DAKO, 1:200), beta-Catenin (14/beta-Catenin, BD Biosciences, 1:50), ARID1A/BAF250a (HPA005456, Sigma Chemicals, 1:200), and γH2AX (Novus Biologicals, 1:1200).

After antigen retrieval, the slides were incubated with the primary antibodies. After incubation for 1 h at room temperature, the staining of ER, PR, beta-Catenin, and γH2AX was further conducted with the Ventana Benchmark automated system (Ventana Medical Systems, USA) using Ventana reagents such as UltraView HRP (for ER, PR), UltraMap™ DAB detection kit for γH2AX, and Optiview (beta-Catenin). The antibodies against the mismatch repair proteins and ARID1A were incubated for 30 min and the staining procedure was carried out with the automated Leica BOND system using the Bond Polymer Refine Detection Kit (Leica Biosystems).

### DNA extraction

Histological slides from FFPE tissue specimens were reviewed for tumour content and the tumour area was marked by a trained gynaecopathologist for tissue punching (Fig. 2). DNA from peripheral blood and FFPE punches (3 cylinders with diameter of 0.6 mm) was isolated with the Maxwell 16 LEV Blood DNA kit (Promega, #AS1290) and Maxwell 16 FFPE Tissue LEV DNA Purification Kit (Promega, #AS1130), respectively, according to the manufacturer’s recommendations. Briefly, 300 μl of blood collected in a BD Vacutainer K2 (EDTA 18.0 mg) tube was added to 30 μl of Proteinase K solution (final concentration 2 mg/ml) and subsequently mixed with 300 μl lysis buffer, vortexed and incubated for 20 min at 56 °C. FFPE cylinders were deparaffinised with xylene, washed twice with ethanol, dried 10 min at 37 °C and resuspended in 200 μl incubation buffer containing 2 mg/ml Proteinase K. Samples were incubated overnight at 70 °C and mixed with 400 μl lysis buffer. Lysates from both, blood and FFPE tissue were transferred to well 1 of the supplied cartridge of the corresponding kit and DNA was automatically purified and eluted in 30 μl Tris-buffer, pH 8.0 by the Maxwell instrument.

### Next generation sequencing (NGS)

Targeted NGS of 409 cancer related genes was performed with 40 ng DNA using the Ion AmpliSeq Comprehensive Cancer Panel (Thermo Fisher Scientific) according to the manufacturer’s protocol. Alignment, variant calling and filtering were performed with Ion Reporter 4.4 (Thermo Fisher Scientific). The following filter chain was used: “Location in utr_3, splicesite_3, exonic, splicesite_5, utr_5” in, “variant effect in stoploss, nonsense, missense, frameshiftInsertion notframeshiftInsertion, nonframeshiftBlockSubstitution, frameshiftDeletion, nonframeshiftDeletion, frameshiftBlockSubstitution” in, “USCS Common SNPs” not in, allele ratio between 0.05 and 1.0 in, allele read-count between 100 and 100 000 in, variant type “INDEL, LONGDEL, SNV, MNV” in. Variants selected after visual inspection of the reads in IGV that displayed more than 15% variant frequency were verified with Sanger sequencing (Additional file 1: Table S1).

### Sanger sequencing

Primers were designed to amplify 200 bp fragments surrounding the position of the detected variant (Additional file 1: Table S1). PCR products were purified and subjected to Sanger sequencing in two reactions, one with the forward and one with the reverse primer.

## Results

### Histopathological considerations

**Fig. 1:**
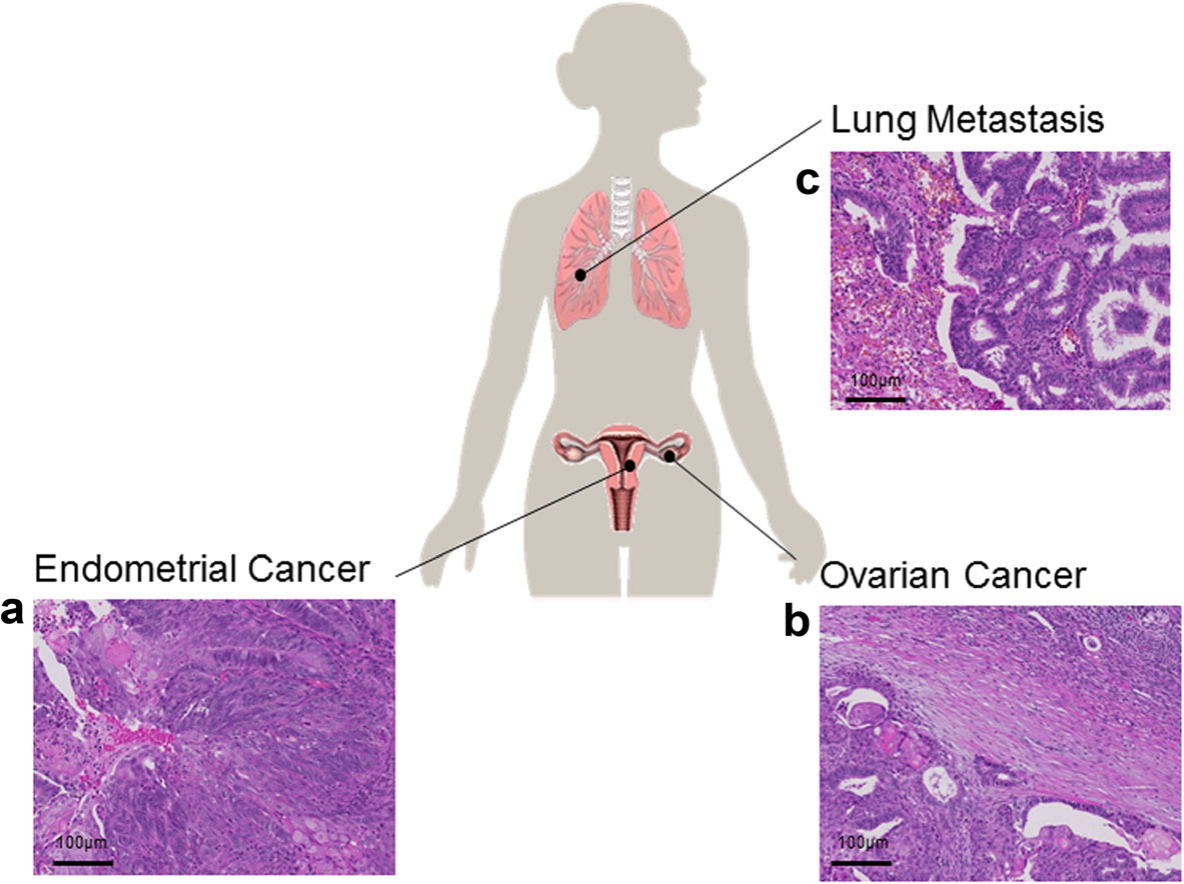
Tumour samples used in the study and representative images of the simultaneous endometrial and ovarian carcinoma as well as the lung metastasis. **a** Endometrioid adenocarcinoma with focal squamous component. **b** Endometrioid adenocarcinoma involving the ovary. On the right upper side an ovarian follicle is present. **c** Endometrioid carcinoma with infiltration of lung tissue. Hematoxylin and eosin staining; original magnification 20x

Histopathological evaluation of the simultaneous endometrial and (unilateral) ovarian cancer as well as the lung metastasis revealed the same tumour morphology, showing an endometrioid adenocarcinoma with low grade features and squamous components (Fig. 1). Squamous differentiation is a common feature of endometrioid type cancer and may occur in both endometrial and ovarian tumours. Precursor lesions like endometrial hyperplasia or components of an ovarian borderline-tumour or endometriosis were not identified.

In summary, the endometrial and ovarian neoplasms showed the very same histomorphology and differentiation, and no precursor lesions were found, neither in the ovary, nor in the uterus. The primary site of cancer origin, however, could not be clarified with this observation. To further address this key question we first compared the size of the lesions. Since the endometrial carcinoma was larger than the ovarian tumour, the uterus is in favour of the primary origin. However, a common or multifocal origin can also be hypothesized.

### Genetic alterations (NGS)

To gain insight into the tumour biology we used targeted deep re-sequencing. DNA was extracted from the tumour area and the correct excision of the FFPE punches was confirmed with HE staining (Additional file 2: Figure S1). 409 genes were re-sequenced with the Ion AmpliSeq Comprehensive Cancer Panel (Thermo Fisher Scientific). Variants present in the blood were excluded (the full table of variants from two technical replicates are listed in Additional file 1: Table S2) and the variants of interest with a frequency of more than 15% were verified with Sanger sequencing (Additional file 2: Figure S2 and data not shown). The variant frequency in each sample was determined as percent variant reads from total reads.

**Fig. 2:**
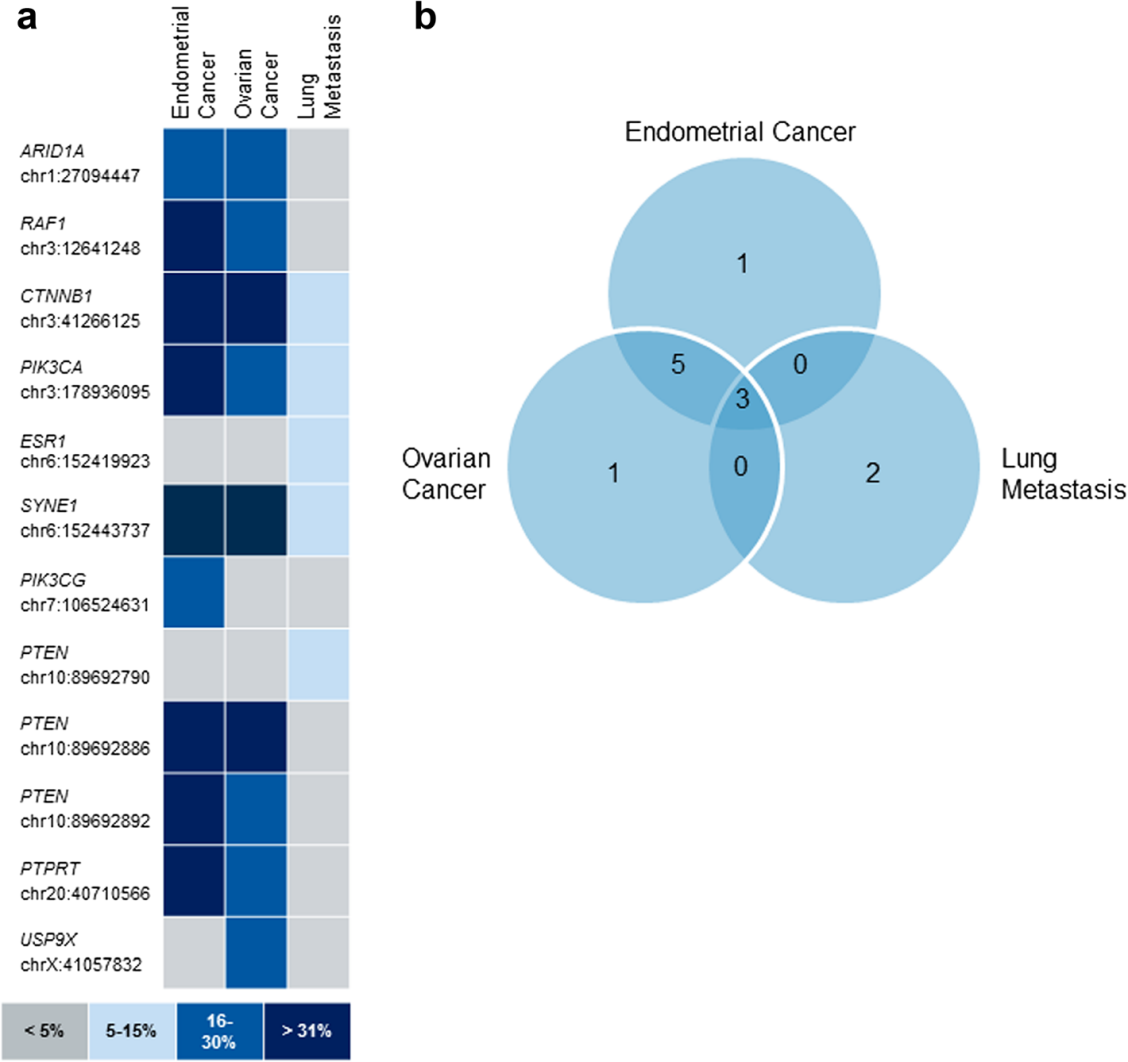
Mutational landscape of the endometrial and ovarian adenocarcinomas and the lung metastasis. **a** Overview of the mutated genes and the positions of the mutations detected with targeted NGS. The intensity of the blue colour encodes for the frequency of the corresponding mutation in each sample (see scale). **b** Venn diagram representation of the number of mutations in each specimen and their overlap. EmCa, endometrial carcinoma; OvCa, ovarian carcinoma; Meta, lung metastasis

Somatic mutations in *ARID1A*, *RAF1*, *CTNNB1*, *PIK3CA*, *ESR1*, *SYNE1*, *PIK3CG PTEN, PTPRT* and *USP9X* were detected (Table 1, Fig. 2a). PhyloP, SIFT, Grantham, PolyPhen are different algorithms predicting the damage caused by a mutation and at least one of them suggested impaired protein function arising from all listed mutations. Furthermore, the SNVs in *CTNNB1*, *PIK3CA*, *PTEN* and *ESR1* were annotated in *COSMIC*, the catalogue of somatic mutations in cancer.

**Table 1:** Mutations, identified with NGS

Gene	Location	Mutation type	Ref - > seq	Blood [%]	EmCa [%]	OvCa [%]	Meta [%]	Coding	Amino Acid Change	PhyloP*	SIFT**	Gr***	PPhen-2****	COSMIC
ARID1A	chr1: 27094447	Insertion	A- > AT	0	30	24	0	c.3155_3156insT	p.Arg1053fs	2.16				
RAF1	chr3: 12641248	Deletion	CA- > C	0	37	30	0	c.1049_1049delT	p.Met350fs					
CTNNB1	chr3: 41266125	SNV	CCA - > TCA	0	35	31	12	c.122C > T	p.Thr41Ile	2.8 0.86 2.26	0	89	0.996	COSM5730; COSM5701; COSM5676; COSM29289
PIK3CA	chr3: 178936095	SNV	A- > G	0	34	29	10	c.1637A > G	p.Gln546Arg	2.2	0.01	43	0.992	COSM767; COSM12459; COSM25041
ESR1	chr6: 152419923	SNV	A- > G	0	0	0	9	c.1610A > G	p.Tyr537Cys	2.02	0	194	0.998	COSM1074637 COSM1074639
SYNE1	chr6: 152443737	SNV	C- > A	0	41	38	6	c.26228G > T	p.Arg8743Ile	2.62		97		
PIK3CG	chr7: 106524631	SNV	C- > G	0	29	0	0	c.2792C > G	p.Ser931Cys	2.6	0	112	1	
PTEN	chr10: 89692790	SNV	T- > A	0	0	0	10	c.274G > T	p.Asp92Tyr	2.32	0	160	1	COSM86049; COSM50762; COSM3566
PTEN	chr10: 89692886	SNV	T- > A	0	32	31	0	c.370 T > A	p.Cys124Ser	2.41 1.95	0.16	112	1	COSM5224; COSM5824; COSM1577271; COSM921089
PTEN	chr10: 89692892	SNV	G- > A	0	34	30	0	c.376G > A	p.Ala126Thr	2.41	0.11	58	0.999	COSM5051; COSM5211; COSM28889
PTPRT	chr20: 40710566	SNV	C- > T	0	32	24	0	c.4285G > A	p.Val1429Met	2.88	0	21	1	
USP9X	chrX: 41057832	SNV	A- > C	0	0	30	0	c.4432A > C	p.Met1478Leu	1.78	0.7	15	0	

* PhyloP score:• Positive scores -- Measure conservation, which is slower evolution than expected, at sites that are predicted to be conserved; • Negative scores -- Measure acceleration, which is faster evolution than expected, at sites that are predicted to be fast-evolving

** SIFT score: • 0.0–0.05 - deleterious; • 0.05–1.0 – tolerated

*** Grantham score: Higher Grantham scores are considered more deleterious

****PolyPhen-2 score: • 0.0–0.15 -- Variants with scores in this range are predicted to be benign; • 0.15–1.0 -- Variants with scores in this range are possibly damaging; • 0.85–1.0 -- Variants with scores in this range are more confidently predicted to be damaging

Interestingly, SNVs in *PTEN* (p.Cys124Ser and p.Ala126Thr) and *PTPRT* (p.Val1429Met), and one-base indels in *RAF1* (p.Met350fs) and *ARID1A* (p.Arg1053fs) were shared by the endometrial and ovarian carcinomas but not found in the lung metastasis. On the other hand, the lung metastasis carried mutations in *ESR1* (p.Tyr537Cys) and *PTEN* (p.Asp92Tyr) that were not detected in the endometrial and ovarian carcinomas. *USP9X* (p.Met1478Leu) was mutated only in the ovarian adenocarcinoma. In contrast, *PIK3CG* (p.Ser931Cys) was altered only in the endometrial adenocarcinoma.

The general distribution of the detected mutations showed that most of them (8/12) are shared by the endometrial and ovarian tumour. Only a single private mutation in the ovarian and endometrial lesion was found (Fig. 2b). Three of eight mutations were also detected in the lung metastasis, although with lower frequencies (Fig. 2a and b). Further, the lung metastasis had acquired two additional mutations.

### Protein expression profile (immunohistochemistry)

**Fig. 3:**
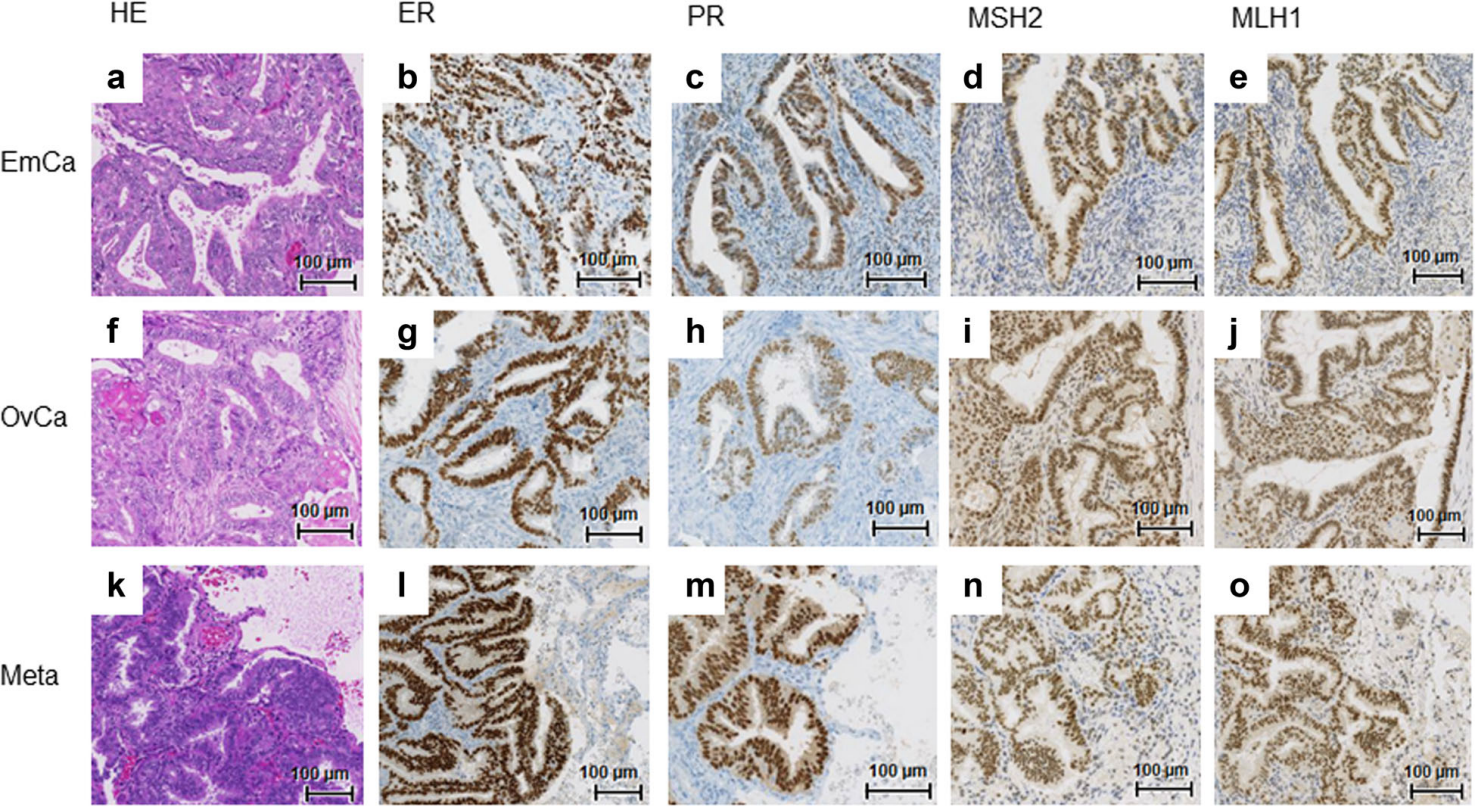
Oestrogen receptors (ER; **b**, **g**, **l**), progesterone receptors (PR; **c**,**h**,**m**) and mismatch repair protein (examples for MSH2 and MLH1; **d**, **i**, **n** and **e**, **j**, **o**) immunohistochemical staining of the endometrial carcinoma (EmCa, **a**-**e**), the ovarian carcinoma (OvCa, **f**-**j**), and of the lung metastasis (Meta, **k**-**o**). Hematoxylin and eosion staining is also shown (**a**, **f**, **k**); original magnification 20x

**Fig. 4:**
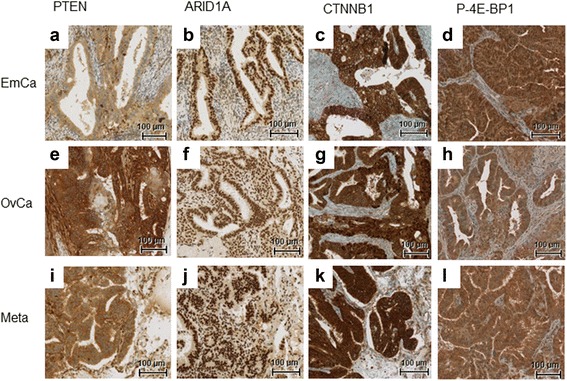
PTEN (**a**, **e**, **i**), ARID1A (**b**, **f**, **j**), CTNNB1 (**c**, **g**, **k**) and P-4E-BP1 (**d**, **h**, **l**) immunohistochemical staining of endometrial carcinoma (EmCa, **a**-**d**), ovarian carcinoma (OvCa, **e**-**h**), and of the lung metastasis (Meta, **i**-**l**); original magnification 20x

The immunohistochemical profile was consistent in all three cancer manifestations. The hormone receptor expression was strong in almost 100% of tumour nuclei. The expression of four mismatch repair proteins (MSH2, MLH1, MSH6, PMS2) was visible in normal and tumour tissue, suggestive of microsatellite stable (MSS) carcinomas (Fig. 3). Expression of PTEN, beta-Catenin, and ARID1A was observed in all tumour samples (Fig. 4). γH2AX staining was used as a surrogate marker for double strand breaks. Nuclear γH2AX expression was observed in single cells of the ovarian tumour but less in the endometrial and metastatic tumour. Interestingly, the expression was found not only in the glandular tumour component but also in the squamous part (data not shown).

## Discussion

In the present study, we investigated the molecular profile of a simultaneous low-grade endometrioid endometrial and ovarian carcinoma as well as of a lung metastasis in a young female patient. From the histology alone, the primary origin is sometimes difficult to determine. The identification of precursor lesions, the pattern of spread and tumour size are valuable parameters for this differentiation. Immunohistochemical analysis of hormone receptors, PTEN and beta-Catenin may show overlapping staining patterns and is therefore not always helpful. The simultaneous occurrence of an endometrial and ovarian carcinoma in women below 50 years of age is a common combination [3], however, the pattern of pulmonary metastasis in this low grade and low stage cancer disease is unusual.

In our molecular analysis, we detected and verified mutations in genes such as *ARID1A*, *CTNNB1*, *PIK3CA,* and *PTEN* which are frequently affected in these tumour entities [7, 8]. However, no striking alterations in mismatch repair genes known to cause early development of endometrial cancer were detected.

We further showed that some mutations were lost during the metastasis formation, either due to the treatment or because of redundancy with other alterations affecting the same pathway. Last but not least, we identified enrichment of mutations causing upregulation of PI3K signalling that could explain the prolonged complete remission under megestrol acetate.

Somatic mutations were identified with targeted PCR-based NGS and verified with Sanger sequencing. The fact that 67% (8/12) of the detected alterations were shared by the ovarian and endometrial tumour with similar variant frequencies suggests a common origin of the two lesions. *PTEN* is more frequently mutated in low-grade endometrial endometrioid carcinomas (67%) compared to low-grade ovarian endometrioid carcinomas (17%). In contrast, *CTNNB1* mutations are significantly different in low-grade ovarian endometrioid carcinomas (53%) compared to 28% of the low-grade endometrial endometrioid carcinomas [4]. In the present case, both genes were mutated in both tumours, hampering the unambiguous identification of the primary lesion. However, all shared mutations showed a slightly higher variant frequency in the endometrial adenocarcinoma. Based on this observation it is tempting to speculate that the endometrial adenocarcinoma gave rise to the ovarian adenocarcinoma. The clones that populated the ovary seemed to be of mixed origin and the cells containing the mentioned mutations could have lost their selection advantage in the ovarian microenvironment. The origin of the lung metastasis, however, could not be specified unambiguously.

The early onset of the malignant disease in this young woman at age 33 suggests possible germline mutations that accelerated the accumulation of genetic aberrations promoting cancer development. A common genetic defect that leads to the development of endometrial cancer in the context of Lynch syndrome is aberrations in the mismatch repair (MMR) proteins. Interestingly, neither mutations in the MMR genes nor increased double strand breaks could be detected. Another possible cause of the early disease onset is Cowden’s syndrome. Targeted PCR-based re-sequencing, however, is not the ideal method to detect large gene deletions. The uniformity blot (sequencing depth across amplicons) for the *PTEN* gene showed no inconsistencies. Therefore, larger deletions on chromosome 10 were unlikely. In addition, germline *PTEN* mutations could be ruled out.

The one base insertion detected in gene *ARID1A* causes a frame shift at codon 1053 of the protein. Since the percentage of the mutation suggested a heterozygous alteration, the sustained immunohistochemical staining is probably due to wild-type ARID1A expressed in the tumour samples. However, our study does not provide information on the expression status of the mutated protein or possible dominant negative interference with wild-type *ARID1A*. The mutation was not present in the metastasis or present at a low percentage from a marginal subclone indicating that this mutation was not substantially contributing to the tumour spread.

In contrast, the somatic mutations in *CTNNB1* and *PIK3CA* were detected not only in the endometrial and ovarian tumours, but also in the lung metastasis. Both mutations are reported in COSMIC, providing experimental evidence that both mutations affect the function of the each protein and are pathogenic. The amino acid change p.Thr41Ile caused by the SNV c.122C > T in *CTNNB1* increases protein stability of beta-catenin and leads to its relocalisation to the nucleus [9]. The SNV c.1637A > G in *PIK3CA* results in a mutant PI3K displaying increased substrate phosphorylation [10]. Hyperactivation of the PI3K pathway is a known cancer driving event in endometrial cancer [8].

Another key protein that is a part of the same signalling cascade and was affected by gene mutations in the described case is the tumour suppressor PTEN. The *PTEN* SNV c.370 T > A results in replacement of the cysteine in the active site against serine (p.C124S) leading to an enzymatically inactive protein. Although, as shown by immunohistochemistry, the protein level was not affected and the mutation was likely to be heterozygous, it has been recently reported that this enzymatically dead mutant inhibits wild-type PTEN in a dominant negative manner [11]. Additionally, we found a second *PTEN* mutation (c.376G > A) that leads to a threonine replacing the alanine at the adjacent position 126 of the protein, likely to distort the tertiary structure of the active site. None of the *PTEN* mutations could be detected in the lung metastasis. Instead, another *PTEN* mutation (p.Asp92Tyr) was detected in the lung metastasis that is also annotated in COSMIC. A possible explanation for the loss of the dominant negative *PTEN* mutation is the biological redundancy of the PTEN inactivating and PIK3CA activating mutations that both lead to an elevated signalling through the PI3K pathway. Indeed, there is evidence that single clones from the primary tumour are able to colonise distant organs and that the metastases carry rather less mutations than the primary tumour due to natural selection process that sustains only the aberrations indispensable for tumour cell survival and proliferation [12]. Interestingly, four years after surgery the patient presented with a lung metastasis that was excised and a therapy with megestrol acetate was initialised. To date, the patient is recurrence-free. Megestrol acetate is reported to act through de-phosphorylation of the PI3K downstream target Akt [13], which might be a possible reason for the complete response of the patient to the treatment.

It has been shown very recently by two independent groups that a majority of synchronous endometrial and ovarian endometrioid carcinomas show clear evidence of clonality [14, 15]. The findings of these two studies strongly indicate that a considerable part of clinically synchronous endometrial and ovarian endometrioid carcinomas constitute in fact dissemination from one site to the other which is also very likely in our case. In our study we extend these results by next generation sequencing analysis of a distant metastasis and demonstrated that not all mutations found in the primary tumour were conserved in the metastasis. This is illustrated e.g. by the SNVs in *PTEN* (p.Cys124Ser and p.Ala126Thr) and *ARID1A* (p.Arg1053fs) which were found in both, the endometrial and the ovarian endometrioid carcinoma, but were not conserved in the distant lung metastasis. Co-occurrence of mutations in *ARID1A* and *PTEN* have been shown as potential cancer driving mutations in endometrioid ovarian carcinomas in animal models [16]. Interestingly, these two mutations were not conserved in the distant metastasis, indicating another clonal event. Neither the *PTEN* nor the *ARID1A* mutation has given rise to the distant metastasis. To our knowledge this is the first case demonstrating the lack of an *ARID1A* mutation in a distant metastasis of an *ARID1A* mutated primary tumour. This demonstrates that even very frequent and potential cancer driving mutations such as *PTEN* and *ARID1A* in endometrioid endometrial and ovarian cancer may not be found in distant metastases, a fact that is essential for potential future targeted therapies [17–19]. However, this also demonstrates the importance of sequencing metachronous distant metastases since its mutational profile may differ from the profile of the primary tumour.

## Conclusions

In conclusion, our results demonstrate that next generation sequencing is an important tool in the differentiation of simultaneous primary tumours and metastases, which has an important impact for clinical practice. This may not only be valuable information for prognostic considerations, but also may be of increasing importance for future targeted therapies.

## Additional files

Additional file 1: Table S1. Sequences of the primers used for Sanger sequencing. **Table S2.** Comprehensive list of variants from targed re-sequencing of blood, ovarian cancer, endometrial cancer and lung metastasis. (xlsx)
Additional file 2: Figure S1. Areas used for DNA extraction in the endometrial (A, B) and ovarian (C, D) carcinoma and the lung metastasis (E, F). The oval gaps in the FFPE tissue correspond to the punches excised from the block and processed for NGS HE staining; EmCa, endometrial carcinoma; OvCa, ovarian carcinoma; Meta, lung metastasis. Figure S2. Electropherograms from Sanger sequencing of *ARID1A*, *CTNNB1*, *PIK3CA* and *PTEN.* Arrows show the peaks of the mutated sites. (PDF)

## Abbreviations

CT: Computer tomography
ER: Oestrogen receptor
FFPE: Formalin fixed paraffin Embedded
Fig: Figure
FIGO: Fédération internationale de gynécologie Obstétrique
MMR: Mismatch repair
MSS: Microsatellite stable
NGS: Next-generation sequencing
PR: Progesterone receptor
PR: Progesterone receptor
Suppl: Supplementary
TCGA: The cancer genome atlas
