# Supplementary material for: Tracking the origin of simultaneous endometrial and ovarian cancer by next-generation sequencing – a case report
Source: BMC Cancer. 2017 Jan 19;17:66. doi: 10.1186/s12885-017-3054-6 (PMC5247816; doi:10.1186/s12885-017-3054-6)
Supplement: Additional file 2: Figure S1. — Areas used for DNA extraction in the endometrial (A, B) and ovarian (C, D) carcinoma and the lung metastasis (E, F). The oval gaps in the FFPE tissue correspond to the punches excised from the block and processed for NGS HE staining; EmCa, endometrial carcinoma; OvCa, ovarian carcinoma; Meta, lung metastasis. Figure S2. Electropherograms from Sanger sequencing of ARID1A, CTNNB1, PIK3CA and PTEN. Arrows show the peaks of the mutated sites. (PDF) [file 12885_2017_3054_MOESM2_ESM.pdf]

Supplementary Information:

Tracking the origin of synchronous endometrial and ovarian cancer by next-generation sequencing – a case report.

Nadejda Valtcheva[1*], Franziska M. Lang[2*], Aurelia Noske[1], Eleftherios P. Samartzis[2], Anna-Maria Schmidt[2], Elisa Bellini[1], Daniel Fink[2], Holger Moch[1], Markus Rechsteiner[1], Konstantin J. Dedes[2*] and Peter J. Wild[1*]

[1]*Institute of Surgical Pathology, University Hospital Zurich, Zurich, Switzerland*
[2]*Department of Gynaecology, University Hospital Zurich, Zurich, Switzerland*

Supplementary Figure 1A-F. Areas used for DNA extraction in the endometrial (A, B) and ovarian (C, D) carcinoma and the lung metastasis (E, F).

Supplementary Figure 2. Electropherograms from Sanger sequencing of *ARID1A*, *CTNNB1*, *PIK3CA* and *PTEN.* Arrows show the peaks of the mutated sites.

Suppl. Table 1. Sequences of the primers used for Sanger sequencing.

Suppl. Table 2. Comprehensive list of variants from targed re-sequencing of the ovarian cancer, endometrial cancer and lung metastasis.

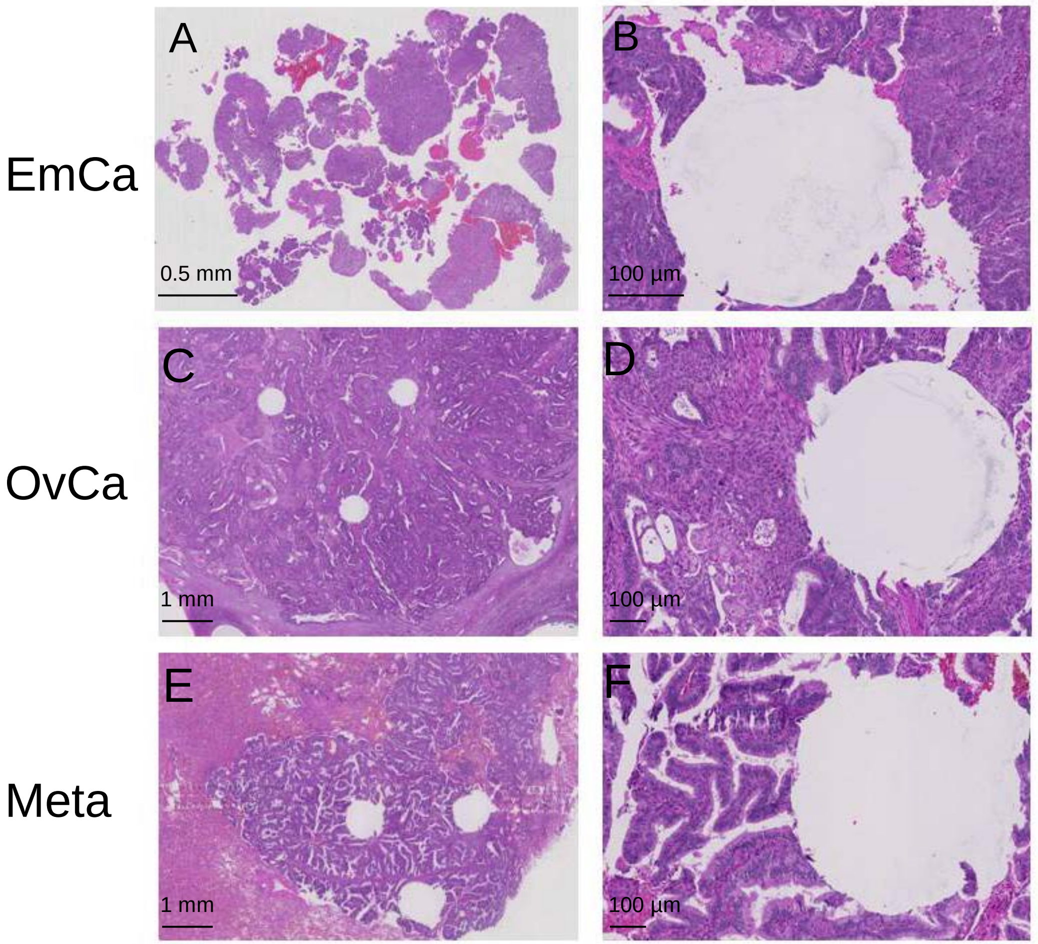

Suppl. Figure 1A-F. Areas used for DNA extraction in the endometrial (A, B) and ovarian (C, D) carcinoma and the lung metastasis (E, F). The oval gaps in the FFPE tissue correspond to the punches excised from the block and processed for NGS HE staining; EmCa, endometrial carcinoma; OvCa, ovarian carcinoma; Meta, lung metastasis.

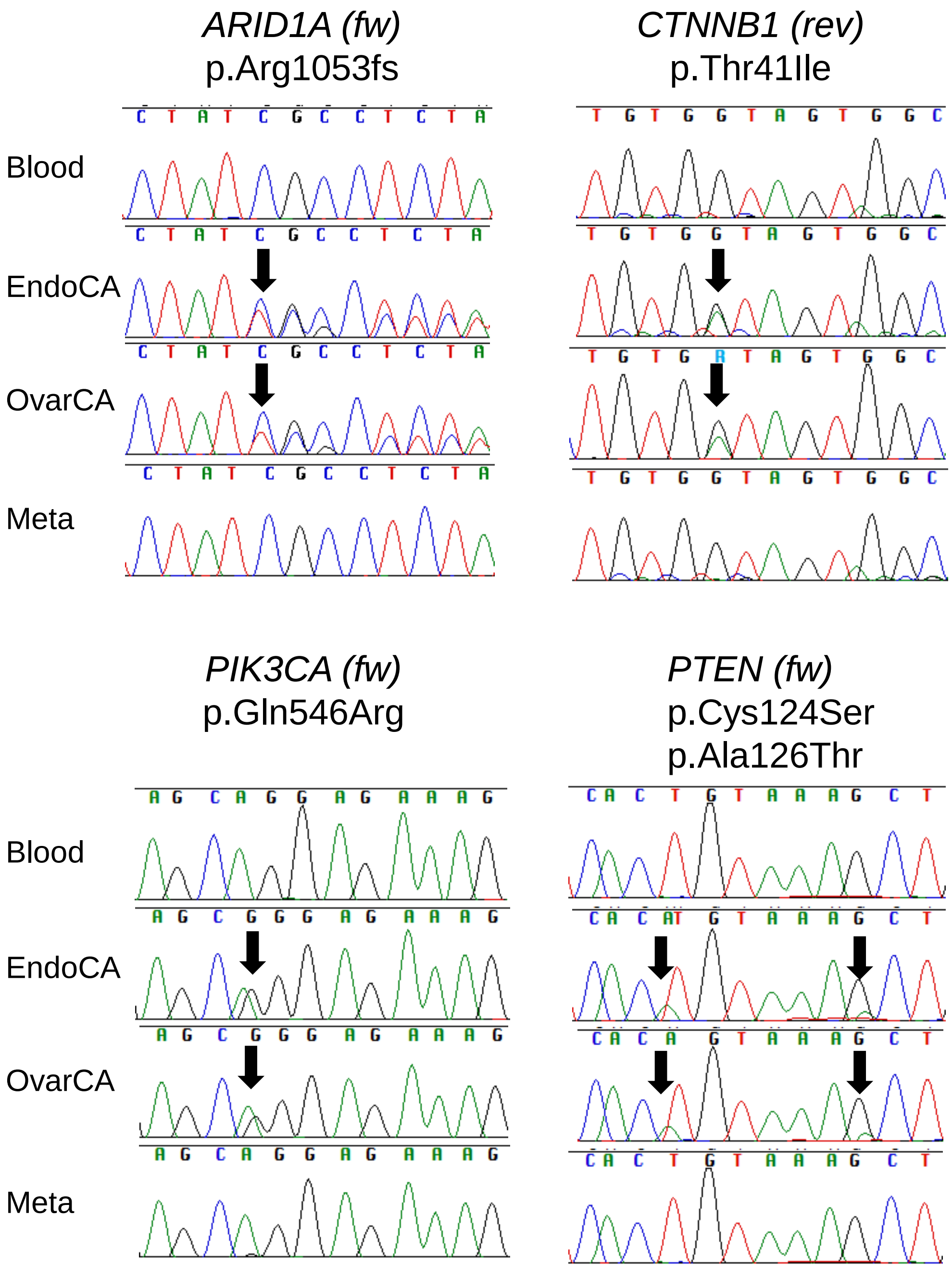

Suppl. Figure 2. Electropherograms from Sanger sequencing of *ARID1A*, *CTNNB1*, *PIK3CA* and *PTEN.* Arrows show the peaks of the mutated sites.

Suppl. Table 1. Sequences of the primers used for Sanger sequencing.

Location	Gene	Forward primer	Reverse primer
chr1:27094447	ARID1A	TTTTGACCTGAACCTTCCAGA	TCACCTGAGTCAATCCACCA
chr3:12641248	RAF1	ATGGGTTGATCCTTTGATGC	ACACCAAAGCCCTGCAGTTA
chr3:41266125	CTNNB1	AAGCGGCTGTTAGTCACTGG	TCAAAACTGCATTCTGACTTTCA
chr3:178936095	PIK3CA	CTGTGAATCCAGAGGGGAAA	TTTAGCACTTACCTGTGACTCCA
chr6:152443737	SYNE1	GAAGGTGCAGAGGCAAAAAG	GGGCACAGCTGTAGTCTTCC
chr7:106524631	PIK3CG	TGCTCTCTGAAAATGGTTTCC	TCACCTGTCTCGGTGATCATA
chr10:89692886/92	PTEN	AGACCATAACCCACCACAGC	TGGTCCTTACTTCCCCATAGAA
chr20:40710566	PTPRT	AGGCCGTAGTGGAACCTTCT	TAGCAGCCAGGTGTTGAGTG
chrX:41057832	USP9X	TCCCTTTTTCAACTTTTTAGGAA	CCTCACACAGCCAACAGCTA

Suppl. Table 2. Comprehensive list of variants from targed re-sequencing of blood, ovarian cancer, endometrial cancer and lung metastasis.
